# A fast and easy one-step purification strategy for plant-made antibodies using Protein A magnetic beads

**DOI:** 10.3389/fpls.2023.1276148

**Published:** 2024-01-03

**Authors:** Loïc Faye, Clemens Grünwald-Gruber, Louis-Philippe Vezina, Véronique Gomord, Bertrand Morel

**Affiliations:** ^1^ ANGANY Innovation, 1 voie de l’innovation, Pharmaparc II, Val de Reuil, France; ^2^ Institute of Biochemistry, Department of Chemistry, Universität für Bodenkultur Wien, Vienna, Austria; ^3^ Angany Inc, St-Jean, QC, Canada

**Keywords:** monoclonal antibody, *Nicotiana benthamiana*, plant molecular farming, magnetic bead purification, COVID - 19

## Abstract

A major difficulty to reach commercial- scale production for plant-made antibodies is the complexity and cost of their purification from plant extracts. Here, using Protein A magnetic beads, two monoclonal antibodies are purified in a one-step procedure directly from non-clarified crude plant extracts. This technique provides significant savings in terms of resources, operation time, and equipment.

## 1 Introduction

Monoclonal antibodies (mAbs) represent 53% of the FDA approvals in the last 4 years (Walsh and Walsh, 2022). More than 80% of these antibodies are expressed in mammalian cells, particularly in Chinese hamster ovary cells (CHOs). Several other expression systems such as bacteria, yeasts, insect cells, and plants have been used for the production of recombinant antibodies or antibody fragments. Today, more than 50 antibodies have been expressed in various plant expression systems. Some of them, such as Caro RX, prevent bacterial tooth decay; the anti-HIV 2G12 or idiotypic scFv for the treatment of non-Hodgkin’s lymphoma have been successfully tested in phase 1 or 2 clinical trials (Ma et al., 2015; Komarova et al., 2019). However, although safety and efficacy of several mAbs produced in plants have been demonstrated and several very large commercial-scale, cGMP-compliant, plant-based production facilities exist in Europe and in North America (Holtz et al., 2015), none of these products are presently commercialized.

One of the major difficulties to reach commercial scale for plant-made pharmaceuticals, including plant-made antibodies, is the complexity and the costs of their purification from plant extracts. For instance, an important issue for downstream processing of plant-made biopharmaceuticals compared to other production systems is the clarification of the plant extract to rapidly remove fibrous particulate, plant pigments, and phenolic compounds. Clarification of plant extract combines centrifugation and filtration by depth and pre-coated filters. Subsequently, their purification implies similar chromatographic techniques.

Magnetic beads have been shown to be versatile tools for easy and effective isolation of biomolecules including genomic DNA, plasmids, RNA, and proteins from bacterial or animal crude cell lysates. Here, a Protein A magnetic bead was used for the purification of plant-made mAbs.

## 2 Materials and methods

### 2.1 Molecular design, cDNA assemblies, and preparation of plasmids

For the expression of the M15 monoclonal antibody (mAb), two constructs were used. The first one encoding for heavy chain (HC) contains HC variable region (accession number: QKQ15189.1) and HC constant region (accession number: AXN93647.1), and the second one encoding for light chain (LC) contains LC variable region (accession number: QKQ15273) and LC constant domain kappa (accession number: P01834.2). Each one was fused at the C-terminus to the tobacco chitinase signal sequence (accession number: QEQ12695). For the expression of ScFV-Fc (S15), the cDNA encoding the light- chain variable region (VL) (accession number: QKQ15273), the heavy- chain variable region (VH) (accession number: QKQ15189.1), and immunoglobulin gamma 1 constant region without CH1 (accession number: AXN93647.1) were fused at the C-terminus to the tobacco chitinase signal sequence (accession number: QEQ12695). ScFv-Fc is in VL-VH orientation, with a (GGGGS)3 linker connecting the VL to the VH.

*Xba* I and *Sal* I restriction sites were, respectively, cloned at the 5′ and 3′ ends of each of the cDNA assemblies described above. These sites were then used to clone the cDNA assembly into the binary expression vector pAG01 (Gomord et al., 2020). pAG01 vector also contained an expression cassette for the silencing inhibitor p19. The vectors were then used to transform *Agrobacterium tumefaciens* strain LBA4404.

### 2.2 Plant cultivation and transient expression

**Figure 1:**
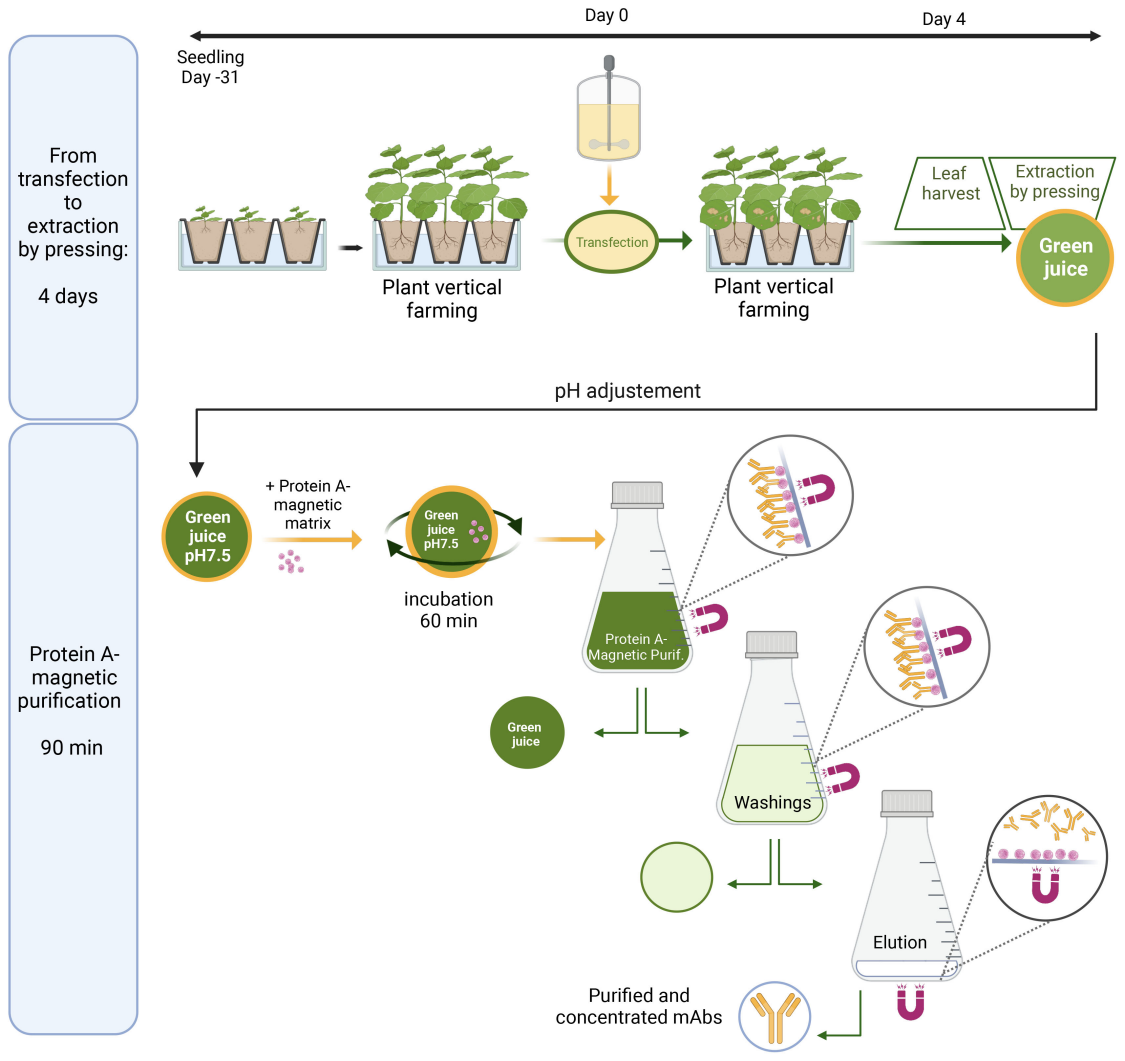
Transient expression and magnetic purification of the plant-made anti-SARS-CoV-2 mAb, M15, and its scFv-Fc homolog S15. Schematic illustration of a M15 and S15 production, from transfection to extraction by tobacco leaf pressing (upper panel) and one-step magnetic purification from crude extracts of *N. benthamiana* (lower panel).

*Nicotiana benthamiana* seeds were sown in coco fiber plugs. As illustrated in **Figure 1**, the seedlings were grown for 14 days in a hydroponic system under continuous LED lighting and then transferred to larger hydroponic tanks containing a nutrient medium under LED lighting at 26°C and a 16 h/8 h day–night regimen where they were allowed to develop for 14 additional days. At the end of this period, their aerial part was immersed in a suspension of agrobacteria carrying the binary vector (the inoculum). The inoculum was then infiltrated in leaves by two cycles of vacuum (−0.8 Bar)/release. Following infiltration, plants were transferred to new hydroponic tanks.

*Nicotiana benthamiana* leaf samples were collected at various time points (3, 4, and 5 days post-infiltration) to determine time-dependent antibody expression levels.

In this study, *N. benthamiana* leaves were harvested 4 days post-infiltration and extracted using a juicer (Angel 5500). After filtration on a combination of nylon filters (300-, 150-, and 30-μ m mesh opening) to remove larger particles, the crude extract is adjusted to pH 7.5 with Tris 1 M (**Figure 1**), and the extract is used for magnetic purification of plant-made mAbs as described below.

### 2.3 Antibody magnetic purification

First, the matrix is prepared by transferring 50 mL of Protein A Mag Sepharose^®^ XTRA (Cytiva) suspension in a 50-mL Corning tube. The tube is placed during 10 s on the magnet, and the storage solution is removed while maintaining the magnet in contact with the edge of the tube. The magnetic bead is washed twice with 50 mL of 50 mM Tris–HCl and 125 mM NaCl buffer, pH 7.5 Tris-buffered saline (TBS), using the same procedure.

Subsequently, 5 mL of the prepared sedimented magnetic matrix is combined with 1 L of green juice and placed in a 1-L Corning storage bottle. Then, the mixture is incubated for 60 min with slow (15 rpm) end-over-end mixing on a rotatory laboratory mixer with fastening systems such as large strips of tape to secure the bottle on a shaker tray. Following incubation, the bottle is placed on the neodynium magnet for 15 min. The green juice supernatant is removed by returning the bottle while maintaining the magnet firmly at its bottom (**Figure 1**). The magnetic matrix is resuspended in 100 mL of TBS, and this suspension is transferred in a large flat bottom 1 L Pyrex beaker. Next, the matrix is washed three times with 100 mL of TBS and two times with 100 mL of TBS/10 (5 mM Tris, 12.5 mM NaCl pH 7.5). For each washing, after matrix resuspension, the beaker is placed 15 s on the magnet before removing the washing buffer while maintaining firmly the magnet at the bottom of the beaker.

Then, the antibody is eluted by the addition of 10 mL elution buffer (100 mM Gly–HCl, pH 2.3) to the magnetic bead. After settling down on top of the magnet for 15 s, the eluted fraction is collected in a separate tube while maintaining firmly the magnet at the bottom of the beaker. This elution procedure was repeated two more times, and 1.2 mL of 2 M Tris was immediately added to the 30 mL fraction containing the purified antibody for neutralization. To preserve the activity of acid-labile antibodies, it is important to work rapidly during antibody elution until adjustment to pH 7.5 with Tris 2 M.

The antibody purity was assessed by SDS polyacrylamide gel electrophoresis (SDS PAGE) and the concentration was determined using quantitation methods such a Bradford assay, ELISA or Biolayer Interferometry.

To regenerate the matrix and to be able to use it for another purification, the magnetic bead is washed twice with 200 mL of 100 mM glycine–HCl buffer, pH 2.3 and then twice with 200 mL of TBS. The matrix is stored at 5°C until further use. It is important to highlight that using this protocol, the same batch of Protein A Mag Sepharose^®^ XTRA was employed for purification of 30 different batches of antibodies without loss of activity.

In the present study, 1.6 –1.8 L of crude extract was typically obtained from 2 kg of tobacco leaves. Thus, starting from 2 kg of tobacco leaves, the purified antibody was obtained in 30 mL of eluate, only 90 min after biomass collection.

### 2.4 SDS-PAGE and Western immunoblot analyses.

Protein samples were heated at 90°C for 10 min in denaturation buffer A (62.5 mM Tris, pH 6.8, containing 10% glycerol, 1% SDS, and 2% β-mercaptoethanol). Then, protein samples were centrifuged at 10,000×*g* for 5 min before loading on gels. SDS-PAGE was performed on 16% or 4%–20% polyacrylamide Tris– glycine gels (Novex WedgeWell, XP0016BOX). For the control of M15 and S15 integrity and purity, electrophoretic separation was performed under reducing or non-reducing conditions, and gels were silver-stained. To study M15 and S15 specificity, following electrophoretic separation, 250 ng of recombinant spike S1 subunit (S1) (Creative diagnostics, DAGC091), 200 ng of RBD (Genscript Z03483) produced in HEK mammalian cells, 500 ng of recombinant S1 (Raybiotech, 230-01101), and 500 ng of recombinant S2 subunit (Raybiotech, 230-01103) produced in *Escherichia coli* were transferred onto a nitrocellulose membrane (Invitrogen iBlot2 gel transfer Device (IB21001) using an Invitrogen iBlot2NC regular stack (IB23001) for immunodetection. M15 or S15 were used as primary antibodies for immunodetection at a concentration of 0.36 µg/mL followed by a secondary mouse anti-human IgG Fc antibody coupled to horseradish peroxidase at a 1:1,000 dilution (Genscript, 50B4A9). Western blots were visualized with Thermo Fisher West Pico Plus ECL detection reagents and an Invitrogen iBright 1500 imaging system.

## 3 Results and discussion

**Figure 2:**
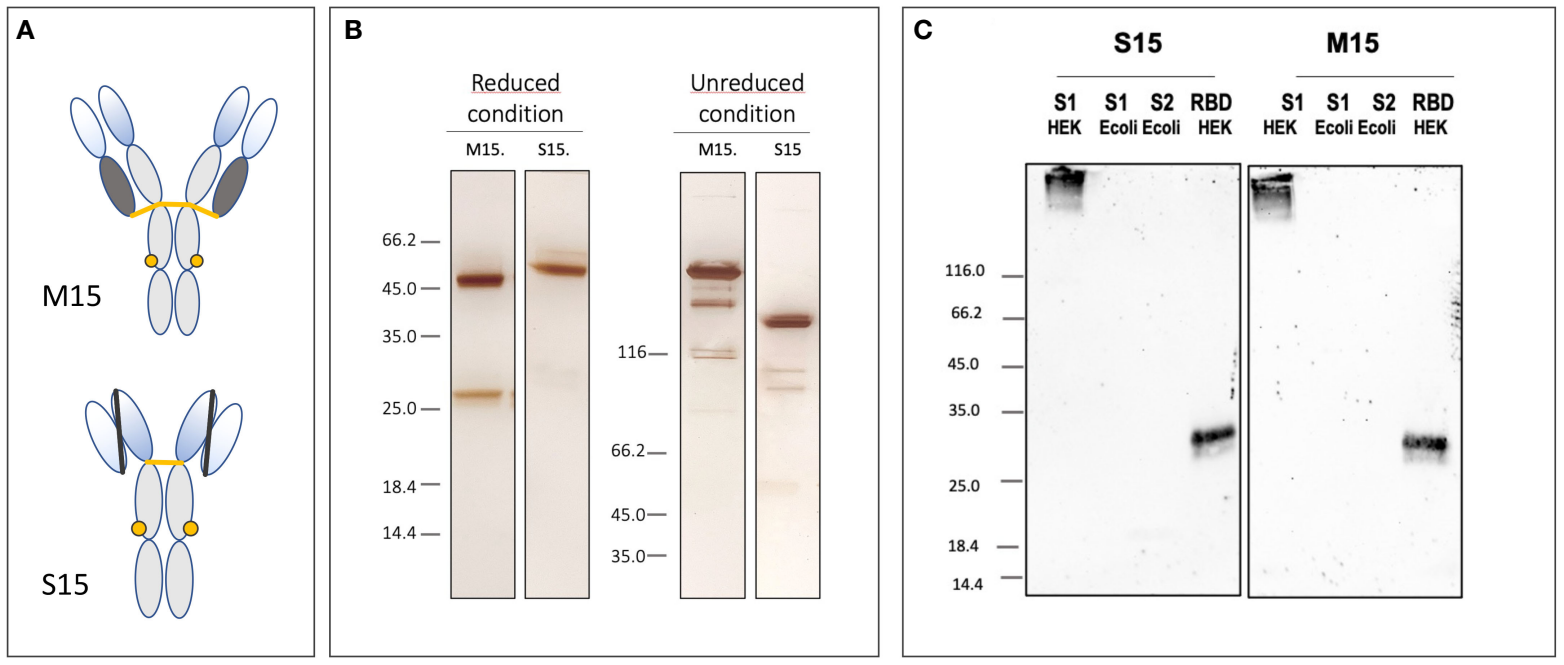
Partial characterization of the plant-made anti-SARS-CoV-2 mAb, M15, and its scFv-Fc homolog S15. **(A, B)** Schematic representation of M15 and S15 and silver-stained SDS-PAGE gels illustrating the integrity and purity of M15 and S15 after transient expression and magnetic purification. **(C)** The specificity of M15 and S15 was evaluated using Western blotting. Both antibodies bind spike protein recombinant S1 subunit and RBD domain produced in HEK293F cells. Spike protein S1 and S2 subunits produced in *E.coli* are not detected by M15 and S15.

A neutralizing antibody binding to Severe accute respiratory syndrome coronavirus 2 (SARS-coV-2) spike protein identified by Brouwer et al. (2020), and its scFv-Fc homolog were codon adapted and transiently expressed in wild- type *N. benthamiana* plants grown at ANGANY’s vertical farming unit. Here, these antibodies are named, respectively, M15 and S15 (**Figure 2A**). Once maximal expression of these antibodies was reached, the green biomass was collected for screw press extraction. In contrast with classical process scheme for monoclonal antibody production in plants where crude extracts centrifugation and filtration are required for clarification before antibody capture step using a Protein A resin, the Protein A magnetic bead purification described here is performed directly from non-clarified crude plant extracts. Therefore, this technique provides significant savings in terms of resources, operation time, and equipment.

Monoclonal antibodies are highly diluted (0.1 –0.2 g/L) in the crude plant extract. This implies large volumes for mAb capture by conventional chromatographic techniques. Indeed, depending on the volume of the applied solution, it can take several hours or even more than a day particularly when antibodies are diluted as it is in plant extracts. In contrast, separation using a Protein A magnetic matrix is a matter of a few hours. Purification time reduction is an important factor for plant-made biopharmaceuticals, as it directly affects the quality of the final product by reducing the contact time of the Abs with phenolic compounds and proteases present in the crude extract. Indeed, a major drawback to antibody production in plants is the loss of material due to proteolytic degradation, and the formation of antibody fragments is frequently described after purification of many plant-made antibodies; for illustration, see Sharp and Doran (2001). Here, due to their fast binding to the magnetic matrix, M15 and S15 are probably rapidly protected from the activity of peptidases released during extraction from tobacco leaves. The quality and purity of these antibodies are illustrated in **Figure 2B** where cleavage products are observed neither for the heavy nor for the light chain of M15 and S15 on SDS-PAGE performed under reducing conditions. Reducing the time needed for mAbs purification using magnetic beads not only improves the quality of the final product but also helps to preserve its biological activity. This is illustrated in **Figure 2C** where the binding capacity of M15 and S15 for the spike protein of SARS-CoV-2 and its RBD domain are studied on blots. M15 and its scFv-Fc homolog S15 are the plant-made versions of COVA2-15, a fully human monoclonal antibody targeting the spike protein of SARS-CoV-2 (Brouwer et al., 2020). After magnetic purification, both plant-made antibodies bind the recombinant S1 subunit of the spike protein and its RBD region being a critical target for mAbs neutralizing SARS-CoV-2. Not surprisingly, M15 and S15 bind the heavily glycosylated S1 subunit expressed in HEK mammalian cells, but S1 produced in *E.coli* is not recognized by these antibodies. From these results, it is anticipated that both M15 and S15 have a similar neutralization potency as COVA2-15 produced in CHO cells. The characterization of the potential of M15 and S15 for passive immunotherapy of COVID-19 was performed both *in vitro* and in a hamster model for coronavirus disease 19 (COVID-19). These results are out of the scope of this Methods article and will be published elsewhere.

The one-step purification presented here not only fuses the steps of clarification and purification but also allows product concentration. Here, starting from 2 L of crude extract, the purified mAb is obtained in 30 mL. As it eliminates intermediate clarification steps, magnetic bead mediated purification also means limited cost in reagents, few materials, and few consumables compared to conventional techniques. Furthermore, only 1 L of buffer is needed for the purification of 2 L starting material.

The use of a Protein A magnetic gel allows purification of antibodies in a one-step procedure from crude plant extracts. Magnetic purification is thus a promising alternative to conventional methods in downstream processing operations for plant-made antibodies, as it eliminates pre-treatment such as centrifugation and filtration and fuses the steps of clarification, purification, and concentration. Magnetic bead purification of plant-made antibodies from crude plant extracts is fast and strongly reduces investments and consumables costs, providing high product purity in a single step.

Considering the advantages of magnetic beads purification, we have adapted this concept to other products such as recombinant allergens fused with an IgG Fc domain or using an Ab covalently linked to a magnetic matrix for immunopurification of an antigen. We also used with success His Mag Sepharose excel™ (Cytiva) for the purification of His-tagged proteins from *N. benthamiana* crude extracts. This further illustrates that magnetic purification is not limited to mAb capture and could be generalized to many other plant-made therapeutic proteins.

## 4 Conclusion and perspectives

In the present study, using a Protein A magnetic bead, mAbs (M15) and its scFv-Fc homolog (S15) has been rapidly purified from non-clarified crude plant extracts, in quantities large enough to study their potential for the inhibition of SARS-COV-2 infection. Some of these results such as N-glycan analysis (**Supplementary Figure S1**; **Supplementary Methods S2**) and binding capacity for SARS-COV-2 spike (S) protein and its receptor binding domain (RBD) are illustrated here. Scaling up of protein magnetic purification is currently under development, and cGMP-compliant equipment for large- scale mAbs purification are already available (Ebeler et al., 2018; Brechmann et al., 2019). This equipment could obviously be adapted for large-scale magnetic purification of plant-made biopharmaceuticals from crude plant extracts. Providing significant savings in terms of resources, operation time, and equipment, magnetic purification should allow a larger and faster diffusion of plant-made mAbs for passive immunotherapy in case of pandemic and facilitate the development of plant molecular farming in developed and less developed countries.

## Data availability statement

The original contributions presented in the study are included in the article/**Supplementary Material**. Further inquiries can be directed to the corresponding author.

## Author contributions

LF: Data curation, Formal analysis, Investigation, Methodology, Writing – original draft. CG-G: Formal analysis, Writing – review & editing. L-PV: Writing – review & editing, Resources. VG: Formal analysis, Conceptualization, Data curation, Investigation, Methodology, Writing – review & editing. BM: Writing – review & editing, Data curation, Formal analysis, Investigation, Methodology.
