# Supplementary material for: A fast and easy one-step purification strategy for plant-made antibodies using Protein A magnetic beads
Source: Front Plant Sci. 2024 Jan 3;14:1276148. doi: 10.3389/fpls.2023.1276148 (PMC10791999; doi:10.3389/fpls.2023.1276148)
Supplement: Supplementary file 1 [file Presentation_1.pdf]

Supplementary Material

FRONTIERS IN PLANT SCIENCE- METHODS

A fast and easy one-step purification strategy for plant-made antibodies using protein A magnetic beads.

Loïc Faye[1*], Clemens Grünwald-Gruber[2], Louis-Philippe Vezina[3], Véronique Gomord[1,3], Bertrand Morel[1]

[1]ANGANY Innovation, 1 voie de l'innovation, Pharmaparc II, 27100 Val de Reuil, France

[2]Institute of Biochemistry, Department of Chemistry, Universität für Bodenkultur Wien, Muthgasse 18, A-1190 Vienna, Austria

[3]ANGANY inc, Suite 200, 873 St-Jean, Québec, QC G1R 1R2, Canada

*Correspondence
Tel: +33 6 08 93 11, loic.faye@angany.com, https://orcid.org/0009-0001-1231-3253

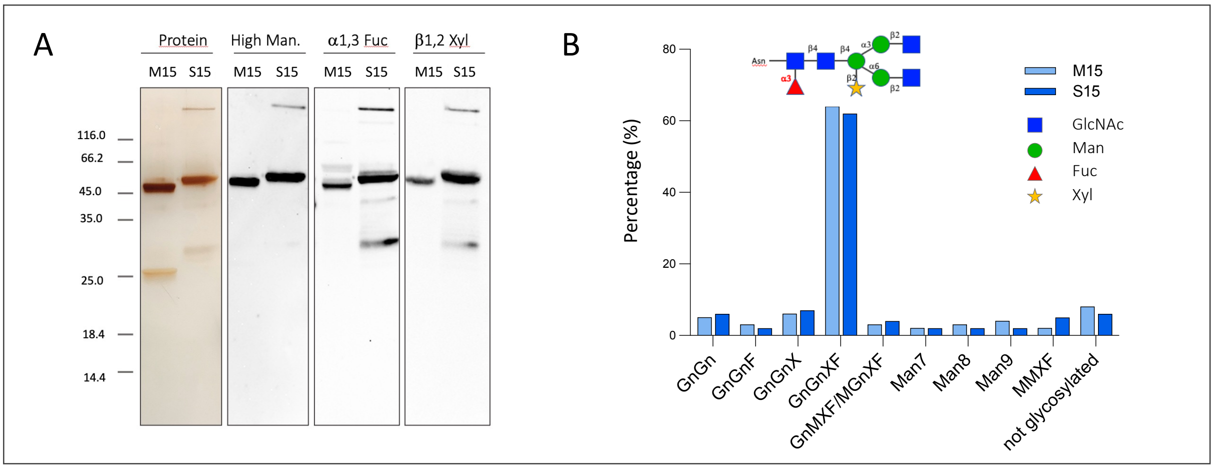

Fig S1. Analysis of M15 and S15 N-glycosylation profiles of blot using N-glycan specific probes (**A**) or by ESI-MS **(B).** The major glycoforms identified were complex type glycans (mainly GnGnXF). Other glycoforms (Man7-Man9, GnGnF, GnGnX, MMXF and GnMXF) were detected as well. Proglycan nomenclature was used for labeling the N-glycans. The detailed N-glycan composition is available in the following link https://homepage.boku.ac.at/jstadlmann/nomenclature.html.

Methods S2: N-linked Glycan analysis

Glycosylation analysis was performed « on blot » for M15 and S15 using antibodies specific for N-linked glycans containing either alpha 1,3 fucose or beta 1,2 xylose residues. These N-glycan-specific probes were prepared and used as previously described (Faye et al., 1993) Mass spectrometry was also used to describe the N-glycosylation profiles of M15 and S15.

These mAbs were digested in-solution, S-alkylated with iodoacetamide and digested with Trypsin (Promega). Additionally, the samples were de-glycosylated using PNGase A to estimate the proportion of not glycosylated peptide and analysed by LC-MS. The digested samples were loaded on a nanoEase C18 column (nanoEase M/Z HSS T3 Column, 100Å, 1.8 µm, 300 µm X 150 mm, Waters) using 0.1 % formic acid as the aqueous solvent. A gradient from 1% B (B: 80% Acetonitrile, 0.1% FA) to 40% B in 50 min was applied, followed by a 10 min gradient from 40% B to 95% B that facilitates elution of large peptides, at a flow rate of 6 µL/min. Detection was performed with an Orbitap MS (Exploris 480, Thermo) equipped with the standard H-ESI source in positive ion, DDA mode (= switching to MSMS mode for eluting peaks). MS-scans were recorded (range: 350-1200 Da) and the 20 highest peaks were selected for fragmentation. Instrument calibration was performed using Pierce FlexMix Calibration

Solution (Thermo Scientific). The possible glycopeptides were identified as sets of peaks consisting of the peptide moiety and the attached N-glycan varying in the number of HexNAc units, hexose, deoxyhexose and pentose residues. The theoretical masses of these glycopeptides were determined with a spread sheet using the monoisotopic masses for amino acids and monosaccharides. Manual glycopeptide searches were made using Freestyle 1.8 (Thermo). For the quantification of the different glycoforms the peak areas of EICs (Extracted Ion Chromatograms) of the first four isotopic peaks were summed, using the quantification software Skyline (University of Washington).

Faye, L., Gomord, V., Fitchettelaine, A.C., Chrispeels, M.J. (1993). Affinity Purification of Antibodies Specific for Asn-Linked Glycans Containing α1 → 3 Fucose or β1 → 2 Xylose. *Analytical Biochemistry* 209, 104-108. doi: 10.1006/abio.1993.1088
